# Predictors for true Actinomyces bacteraemia

**DOI:** 10.1099/jmm.0.002026

**Published:** 2025-06-04

**Authors:** Mamta Sharma, Susan Szpunar, Farah Tanveer, Jonathan Arcobello, Sanjay Revankar, Ashish Bhargava

**Affiliations:** 1 Division of Infectious Disease, Henry Ford St John Hospital, Detroit, Michigan, USA; 2 Department of Biomedical Investigations and Research, Detroit, Michigan, USA; 3 Division of Infectious Disease, Detroit Medical Center/Wayne State University, Detroit, Michigan, USA; 4 Thomas Mackey Center for Infectious Disease Research, Detroit, Michigan, USA

**Keywords:** *Actinomyces* bacteraemia, mortality associated with *Actinomyces*, risk factors associated with *Actinomyces*

## Abstract

**Introduction.** *Actinomyces* species colonizing the human oropharynx and gastrointestinal and urogenital tract are associated with a wide range of infections. The isolation of *Actinomyces* spp. from sterile clinical samples is regarded as significant.

**Gap Statement.** Increased use of advanced diagnostics has caused an increased detection of *Actinomyces* in the bloodstream, the clinical significance of which is unclear.

**Aim.** To investigate the clinical factors associated with true *Actinomyces* bacteraemia that could aid in differentiating it from transient *Actinomyces* bacteraemia.

**Methodology.** We conducted a retrospective study of all inpatients with *Actinomyces* bacteraemia from two tertiary care centres from 1 January 2006 to 26 September 2021. Data were collected on demographic and clinical characteristics, comorbidities, primary source of infection and outcomes. True bacteraemia was defined as *Actinomyces* bacteraemia with systemic manifestations of infection.

**Results.** A total of 82 cases of positive blood cultures were identified, of which 33 (40.2%) were true bacteraemia, based on clinical criteria. Patients with true bacteraemia were more likely to be older (*P*=0.007), have chronic skin ulcers (*P*<0.001), have a history of central line placement within 3 months of their presentation (*P*=0.04), have had a fever within 72 h of admission (*P*=0.05) and have presented with an abscess (*P*<0.001) compared with patients with transient bacteraemia. True bacteraemia was more likely to be associated with positive tissue cultures (*P*=0.02) and an infectious disease consultation than transient bacteraemia. Skin and soft tissue (27.3%) was the most common source followed by intra-abdominal (21.1%). Among true bacteraemia, the most common species was *Actinomyces meyeri* with a ratio of 1:8 (transient versus true bacteraemia). All-cause mortality was 30.3% in patients with true bacteraemia compared with 4.1% in patients with transient bacteraemia (*P*<0.001).

**Conclusion.** Predictors of true *Actinomyces* bacteraemia included older age, fever within 72 h of admission, presence of abscess and chronic skin disease. *Actinomyces* species exhibit varying degrees of invasiveness, with *A. meyeri* potentially showing higher invasive potential. Better awareness and involvement of infectious disease specialists is recommended in determining the clinical significance of transient *Actinomyces* bacteraemia and can help implement antibiotic stewardship and patient safety and improve outcomes. Further research will help to identify the true importance of these isolates.

## Introduction

*Actinomyces* species are filamentous Gram-positive bacilli, mainly belonging to the human commensal flora of the oropharynx, gastrointestinal tract and urogenital tract [1]. *Actinomyces* species can cause actinomycosis, a chronic granulomatous lesion, forming suppurative abscesses and draining sinuses. The most common sites of infection are cervico-facial (50%), abdominal (20%) and thoracic (15%); the remainder are pelvic and cutaneous (15%) [2]. Of note, in any site, actinomycosis frequently mimics malignancy, tuberculosis or other mycobacterium, as it spreads continuously and progressively and often forms a cold abscess [3,6]. More than 30 species of *Actinomyces* have been described. *Actinomyces israelii* is the most prevalent species isolated in human infections and is found in most clinical forms of actinomycosis [7]. Some other species, including *Actinomyces odontolyticus*, *Actinomyces gerencseriae* (formerly *A. israelii* serotype 2), *Actinomyces meyeri*, *Actinomyces turicensis*, *Actinomyces europaeus* and *Actinomyces cardiffensis*, have been associated with disseminated cases with or without bacteraemia [8,15].

Historically, the isolation of *Actinomyces* spp. from sterile clinical specimens has always been considered important [16]. With the use of newer technologies such as matrix-assisted laser desorption ionization-time-of-flight (MALDI-TOF) MS, however, an increase in the isolation of *Actinomyces* spp. from blood cultures has been reported [17]. In a retrospective study of 60 patients with *Actinomyces* bacteraemia, only 10 received treatment for actinomycosis. Patients with transient bacteraemia without any treatment did not show any apparent negative impact on clinical outcomes [17]. Thus, the isolation of *Actinomyces* spp. from blood cultures of patients without clinical disease raises the question of whether these organisms are contaminants or represent transitory bacteraemia due to translocation. An understanding of the factors that can help differentiate between true and transient bacteraemia episodes will be helpful in disease management.

This study investigated the clinical factors associated with true *Actinomyces* bacteraemia that could aid in differentiating it from transient *Actinomyces* bacteraemia.

## Methods

This study was a historical cohort study of all inpatients with *Actinomyces* bacteraemia from two academic tertiary care centres from 1 January 2006 to 26 September 2021. Data were collected by retrospective chart review. Cases of *Actinomyces* bacteraemia were identified using ICD-9 and ICD-10 codes, respectively, for actinomycosis (039.9 and A42.9), bacteraemia (790.7 and R78.81) and sepsis (995.91/995.92 and R65.20/R65.21/A41.4/A42.7). We reviewed microbiology records to confirm the presence of *Actinomyces* bacteraemia in all patients.

Before 2015, the RapID ANA II Identification system was used to identify *Actinomyces* bacteraemia, or samples were sent to a reference lab. Since 2015, the MALDI-TOF MS analyser has been utilized.

The study was approved by the Institutional Review Board (IRBNET number RMIJ20210317).

### Data collection

The following data elements were recorded for each patient with *Actinomyces* bacteraemia: demographics, comorbid conditions, clinical characteristics, implicated source, *Actinomyces* species and treatment given.

### Definitions

True bacteraemia was defined as *Actinomyces* bacteraemia with systemic signs of infection.

Transient bacteraemia was defined as *Actinomyces* bacteraemia without systemic manifestations of infection. Sources of bacteraemia included oropharyngeal, cutaneous, respiratory tract, endovascular source (aneurysms, vascular grafts or other endovascular devices), endocarditis, osteomyelitis, miscellaneous and unknown (systemic signs of infection without an obvious source or in the presence of multiple potential sources). To account for the burden of comorbidities, the Charlson weighted index of comorbidity was computed. Immunosuppressed status included human immunodeficiency virus positivity, neutropenia with absolute neutrophil counts less than 500 cells per microliter at the time of presentation, receipt of steroids (equivalent to at least 16 mg of prednisone per day for at least 5 days consecutively), a fever in the prior 72 h or at the time of presentation, receipt of chemotherapy or radiotherapy or anti-tumour necrosis factor-*α* therapy in the past 3 months and history of bone marrow or solid organ transplant. Renal impairment was defined as mild if serum creatinine was 1.5–2-fold higher than baseline or severe if it were 2-fold higher than baseline or if the patient received haemodialysis or peritoneal dialysis.

### Statistical methods

Descriptive statistics were generated to characterize the study population. Continuous variables were described as the mean with sd or median with interquartile range. The association between clinical and demographic factors and outcomes was assessed using Student’s t-test and ANOVA for the comparison of means and the chi-square test for categorical variables. Multivariable analysis was done using logistic regression. All data were analysed using SPSS v. 29.0, and a *P*-value of 0.05 or less was considered to indicate statistical significance. The study was approved by the Ascension Institutional Review Board.

## Results

In this study, 82 cases of positive blood cultures with *Actinomyces* were identified. Of these, 40.2% (33) were diagnosed with true bacteraemia, whereas transient bacteraemia was diagnosed in 59.8% (49). The mean (sd) age of 82 patients with bacteraemia was 54.9 (±27.2) years; 54.9% (45) were female, and 76.8% (63) were black/African American. Common comorbid conditions in patients included diabetes mellitus (DM) (29.3%), myocardial infarction (MI) (24.4%), chronic obstructive pulmonary disease (COPD) (20.7%), congestive heart failure (CHF) (14.6%) and cerebrovascular disease (14.6%). Chronic skin ulcers were noted in 18.3% of patients, while 12.2% had immunosuppression. A history of hospitalization and invasive procedures within 90 days of bacteraemia was noted in 32.9% and 28% of patients, respectively. Within 3 months of bacteraemia, 9.8% of patients had a central line placed.

Patients with true bacteraemia had a higher mean age than those with transient bacteraemia, 63.9±19.5 versus 48.8±30.0 years, respectively (*P*=0.007). Patients with true bacteraemia were more likely to have chronic skin ulcers (*P*<0.001), a history of central line placement within 3 months of their presentation (*P*=0.04), a fever within 72 h of admission (*P*=0.05) and to have presented with an abscess (*P*<0.001) compared with patients with transient bacteraemia. True bacteraemia was more likely to be associated with positive tissue cultures (*P*=0.02) and an infectious disease consultation (*P*<0.001) than transient bacteraemia (Table 1). All-cause mortality was 30.3% in patients with true bacteraemia compared with 4.1% in patients with transient bacteraemia (*P*<0.001). The most common sources of infection in true bacteraemia were skin and soft tissue 27.3% (9), followed by intra-abdominal 21.1% (7), osteomyelitis 15.2% (5), respiratory 12.1% (4), odontogenic 6.1% (2), endovascular 6.1% (2), endocarditis 3% (1) and genitourinary 3% (1). In 6.1% (2) of cases, no specific source of infection was identified.

**Table 1.** Univariable analysis of factors associated with true *Actinomyces* bacteraemia

Characteristic	Transient bacteraemia (*n*=49)	True bacteraemia (*n*=33)	*P*-value
Age in years (mean±sd)	48.8±30.0	63.9±19.5	0.007
Sex			
Male	23 (46.9)	14 (42.4)	0.69
Female	26 (53.1)	19 (57.6)	
Race			
White	8 (16.3)	5 (15.2)	0.39
Black	39 (79.6)	24 (72.7)	
Others	2 (4.1)	4 (12.1)	
Median CWIC (range)	2 (0, 12)	2 (0, 12)	0.24
MI	12 (24.5)	8 (24.2)	0.98
CHF	7 (14.3)	5 (15.2)	0.91
COPD	11 (22.4)	6 (18.2)	0.64
Peripheral vascular disease	2 (4.1)	5 (15.2)	0.08
DM without complications	7 (14.3)	7 (21.2)	0.41
DM with complications	5 (10.2)	5 (15.2)	0.50
Dementia	4 (8.2)	5 (15.2)	0.32
Cerebrovascular disease	6 (12.2)	6 (18.2)	0.46
Hemiplegia	4 (8.2)	3 (9.1)	0.88
Peptic ulcer disease	4 (8.2)	6 (18.2)	0.17
Mild liver disease	3 (6.1)	1 (3.0)	0.52
Severe liver disease	0	1 (3.0)	
Haemodialysis	5 (10.2)	3 (9.1)	0.87
Immunosuppressed status	7 (14.3)	3 (9.1)	0.48
Chronic skin ulcer	3 (6.1)	12 (36.4)	<0.001
Active malignancy	6 (12.2)	4 (12.1)	0.99
Metastatic disease	4 (8.2)	2 (6.1)	0.72
Recent hospitalization in the last 3 months	17 (34.7)	10 (30.3)	0.68
Invasive procedures in the last 3 months	13 (26.5)	10 (30.3)	0.71
Dental procedures in the last 3 months	0	1 (3.0)	
Central lines in the last 3 months	2 (4.1)	6 (18.2)	0.04
Rash	2 (4.1)	2 (6.1)	0.68
Swelling or skin mass	3 (6.1)	3 (9.1)	0.61
Fever within 72 h of admission	16 (32.7)	18 (54.5)	0.05
Timing of positive blood culture			0.70
Within first 48 h	40 (81.6)	28 (84.8)	0.26
After 48 h	9 (18.4)	5 (15.2)	0.006
Polymicrobial	19 (38.8)	16 (48.5)	0.38
Infectious disease consultation	19 (38.8)	26 (78.8)	<0.001
Abscess	2 (4.1)	10 (30.3)	<0.001
Wound cultures	1 (2.1)	4 (12.5)	0.06
Tissue cultures	2 (4.1)	7 (21.2)	0.02

CWIC, Charlson weighted index of comorbidity; n, Number; SD, Standard deviation.

In 84.8% (28) cases of true bacteraemia, *Actinomyces* were isolated to the species level (Table 2). The most common species was *A. meyeri* 24.2% (8) followed by *A. odontolyticus* 21.2% (7), *A. israelii* 21.2% (7), *A. turicensis* 12.1% (4) and *Actinomyces neuii* 6.1% (2).

**Table 2.** *Actinomyces* species isolated by cases

*Actinomyces* species	Total case (*n*=82) (%)	Transient bacteraemia (*n*=49) (%)	True bacteraemia (*n*=33) (%)	Ratio of transient bacteraemia to true bacteraemia
*A. odontolyticus*	29 (35.4)	22 (44.9)	7 (21.2)	1:0.32
*A. israelii*	16 (19.5)	9 (18.4)	7 (21.2)	1:0.78
*Actinomyces* undifferentiated	13 (15.9)	8 (16.3)	5 (15.2)	1:0.62
*A. meyeri*	9 (11)	1 (2)	8 (24.2)	1:8
*A. neuii*	6 (7.3)	4 (8.2)	2 (6.1)	1:0.5
*A. turicensis*	5 (6.1)	1 (2)	4 (12.1)	1:4
*Actinomyces oris*	3 (3.7)	3 (6.1)	0	
*Actinomyces naeslundii*	1 (1.2)	1 (2)	0	

*n*, Number.

In cases of true bacteraemia, *A. meyeri* was the most common species, with a ratio of transient bacteraemia to true bacteraemia of 1:8. On the other hand, *A. odontolyticus* was the most common species among transient bacteraemia, with a ratio of 1:0.32. No cases of *Actinomyces oris* and *Actinomyces naeslundii* were seen among true bacteraemia (Table 2).

On multivariable analysis, predictors of true bacteraemia included older age (*P*=0.003), fever within 72 h of admission (*P*=0.024), presence of abscess (*P*=0.007) and chronic skin disease (*P*=0.003) (Table 3).

**Table 3.** Multivariable analysis of factors associated with true *Actinomyces* bacteraemia

Variable	Odds ratio (95% confidence interval)	***P*-value**
Age	1.06 (1.02–1.09)	0.003
Fever within 72 h of admission	4.11 (1.21–13.98)	0.024
Abscess	15.31 (2.09–111.96)	0.007
Chronic skin ulcers	11.34 (2.24–57.56)	0.003

## Discussion

In our study cohort, the incidence of true *Actinomyces* bacteraemia was 40.2%. Previous studies have reported that the incidence of *Actinomyces* bacteraemia varies between 16.7% and 80%. An earlier study found that the incidence of *Actinomyces* bacteraemia was lower than ours, at 16.7%, based on 60 cases. In this historical cohort study, only 10 out of 60 cases were treated with antibiotics because of identified clinical risk factors. The use of antibiotics in these instances may serve as a surrogate indicator of actual bacteraemia. In contrast, we defined true *Actinomyces* bacteraemia based on the systemic manifestations of infection at the time of their presentation [17]. The other two studies that reported higher incidences of *Actinomyces* bacteraemia than our study were case series. They reported cases of *A. odontolyticus* bacteraemia, with 76.2% (16/21) and 80% (12/15) of the patients receiving antibiotics [18,19].

Our study findings also included independent predictors of true *Actinomyces* bacteraemia: older age, fever within 72 h of admission, presence of abscess and chronic skin ulcers. Our study is the first clinical study to compare patients with true versus transient *Actinomyces* bacteraemia to assess the factors associated with true *Actinomyces* bacteraemia. Most prior studies identifying the risk factors for *Actinomyces* bacteraemia were based on case reports or case series [19]. Another study assessed the risk factors associated with serious *Actinomyces* bacteraemia in patients who were treated for the bacteremic episodes versus those who were not treated. Unlike our study, the above study did not assess the systemic signs of infections at the time of *Actinomyces* bacteraemia [17].

Our study found that age is an independent risk factor for *Actinomyces* bacteraemia. Every 1-year increase in age increased the risk of *Actinomyces* bacteraemia by 6%. In a previous case series of *A. odontolyticus* bacteraemia, approximately one-third of the reported cases involved individuals aged 60 years and older [20]. Four out of seven elderly patients in this study were immunosuppressed. One study of *Actinomyces* bacteraemia found a median age of 57 years [21]. In another case series on actinomycosis, the median age reported was 67 years [22]. Earlier studies have demonstrated that the severity of bacteraemia increases with age, necessitating quicker identification of organisms and prompt empirical antibiotic treatment [23].

Our study utilized clinical criteria at admission to identify patients with true bacteraemia. We found that fever within 72 h of hospitalization was significantly higher among patients diagnosed with true bacteraemia than those with transient bacteraemia. A previous study reported that fever was present in 80% of patients who required antibiotic treatment for *Actinomyces* bacteraemia [19]. Fever accompanied by systemic manifestations prompts providers to suspect sepsis and initiate empiric antibiotic treatments, which help reduce the incidence of secondary adverse outcomes [19,23, 24].

Disruption of mucosa enables entry of *Actinomyces* to gain access to deeper tissues via trauma, surgical procedures or foreign bodies. Actinomycosis is characterized by a dense lesion that slowly spreads ignoring tissue planes. Over time, fluctuation and suppuration develop. Depending on location over time, sinus tracts can extend from the abscess to the skin or adjacent organs.

In our study, chronic skin ulcers and abscesses were significantly higher in patients with true bacteraemia. In a previous study, skin and soft tissue infection was seen in 30% of cases of true bacteraemia [17]. In another study, 8.8% of cases had skin and soft tissue involvement [21].

The most common species among true bacteraemia was *A. meyeri*, and among the transient bacteraemia was *A. odontolyticus*, with a transient versus true bacteraemia ratio of 1:8 and 1:0.32*,* respectively.

In another study among invasive disease in cancer patients, *A. odontolyticus* was most frequently isolated (35.4%), followed by *A. meyeri* in 20 (20.8%) and *A*. *naeslundii* in 20 (20.8%). In contrast, among colonizer, *A. odontolyticus* (46.2%) was the most frequently isolated species [25]. Based on our study, *A. meyeri* may exhibit higher invasiveness potential, which could guide clinicians to consider aggressive therapeutic measures earlier.

The strength of our study included a multicentre study design. The main limitation of our study was that the data were collected by retrospective chart review. Despite this limitation, we made every effort to ensure the accuracy and reliability of our findings. Our study still provides valuable insights and highlights the need for further research. Second, the study included patients from the two hospitals in Southeast Michigan, so the findings may not be generalizable.

## Conclusion

Predictors of true *Actinomyces* bacteraemia included older age, fever within 72 h of admission, presence of abscess and chronic skin disease. Better awareness and involvement of infectious disease specialists is recommended in determining the clinical significance of transient *Actinomyces* bacteraemia and can help implement antibiotic stewardship to improve appropriate antibiotic use and patient safety and improve outcomes. *Actinomyces* species exhibit varying degrees of invasiveness, with *A. meyeri* potentially showing higher invasive potential in our study, which could guide clinicians to consider aggressive therapeutic measures earlier. Further research will help to identify the true importance of these isolates.

## Abbreviations

CHF: congestive heart failure
COPD: chronic obstructive pulmonary disease
DM: diabetes mellitus
MALDI-TOF: matrix-assisted laser desorption ionization-time-of-flight
MI: myocardial infarction
